# Prevalence and types of rectal douches used for anal intercourse: results from an international survey

**DOI:** 10.1186/1471-2334-14-95

**Published:** 2014-02-21

**Authors:** Marjan Javanbakht, Shauna Stahlman, Jim Pickett, Marc-André LeBlanc, Pamina M Gorbach

**Affiliations:** 1 Department of Epidemiology, Fielding School of Public Health, University of California, 90095-1772 Los Angeles, CA, USA; 2 International Rectal Microbicide Advocates, 411 South Wells Street, Suite 300, IL 60607 Los Angeles, Chicago, USA

**Keywords:** Rectal health, Rectal douching, Enema use, Anal intercourse

## Abstract

**Background:**

Rectal products used with anal intercourse (AI) may facilitate transmission of STIs/HIV. However, there is limited data on rectal douching behavior in populations practicing AI. We examined the content, types of products, rectal douching practices and risk behaviors among those reporting AI.

**Methods:**

From August 2011 to May 2012, 1,725 women and men reporting receptive AI in the past 3 months completed an internet-based survey on rectal douching practices. The survey was available in English, French, German, Mandarin, Portuguese, Russian, Spanish, and Thai and included questions on sexual behaviors associated with AI including rectal douching. Differences by rectal douching practices were evaluated using chi-square methods and associations between reported douching practices and other factors including age and reported STI history were evaluated using logistic regression analysis.

**Results:**

Respondents represented 112 countries, were mostly male (88%), and from North America (55%) or Europe (22%). Among the 1,339 respondents (66%) who reported rectal douching, most (83%) reported always/almost always douching before receptive AI. The majority of rectal douchers reported using non-commercial/homemade products (93%), with water being the most commonly used product (82%). Commercial products were used by 31%, with the most common product being saline-based (56%). Rectal douching varied by demographic and risk behaviors. The prevalence of rectal douching was higher among men (70% vs. 32%; p-value < .01), those reporting substance-use with sex (74% vs. 46%; p-value < .01), and those reporting an STI in the past year (69% vs. 57% p-value < .01) or ever testing HIV-positive (72% vs. 53%; p-value < .01). In multivariable analysis, adjusting for age, gender, region, condom and lubricant use, substance use, and HIV-status, douchers had a 74% increased odds of reporting STI in the past year as compared to non-douchers [adjusted odds ratio (AOR) = 1.74; 95% CI 1.01-3.00].

**Conclusion:**

Given that rectal douching before receptive AI is common and because rectal douching was associated with other sexual risk behaviors the contribution of this practice to the transmission and acquisition of STIs including HIV may be important.

## Background

Anal intercourse (AI) without condoms represents one of the most efficient modes of sexual transmission of HIV [1] and is a risk factor for the transmission of other sexually transmitted infections (STIs). A number of studies have raised concerns about the potential for rectal products used with AI to facilitate transmission of STIs including HIV. The COL-1492 trial provided evidence that vaginal application of Nonoxynol-9 (N9) was associated with increased risk of HIV infection, and further studies showed that rectal administration of N9 was associated with sloughing of rectal epithelia [2-4]. Furthermore, *in vitro* and animal studies have demonstrated that some commercial lubricants may damage rectal tissue [5-9]. In a clinical study, lubricant products caused short-term denudation of rectal epithelium, which was suggested to be induced by the lubricant’s osmotic effect on the rectal mucosa [10]. Cell contact with hyperosmolar solutions (like many lubricants) can cause cells to dry up and collapse. Such injury of the rectal epithelia has been hypothesized to enhance the probability of transmission of pathogens such as HIV [10] and other STIs. In addition to biologic plausibility, a recent epidemiologic study demonstrated that lubricant use during AI was independently associated with rectal STIs [11].

Other practices that may affect the rectal epithelium and enhance STI/HIV transmission include the use of rectal douches and enemas. Vaginal douching has long been associated with a number of STIs, such as chlamydia and gonorrhea [12-15]. Likewise, a number of studies have demonstrated an association between the use of rectal douches/enemas and HIV [16-19], though data on the association with other STIs is limited, with one study showing an association with Lymphogranuloma venereum (LGV) proctitis [20] and another with Hepatitis B virus (HBV) [21]. A recent survey of men who have sex with men (MSM) in the United States found that 44-53% reported rectal douching before last receptive AI [22], with the prevalence as high as 64% in the past 6-months [18]. Additionally, a study among Peruvian MSM found that 27% reported a history of rectal douching [23]. However, little is known about the specific content and types of douches used. Because the prevalence of rectal douching may be relatively high and the products used may cause damage to the rectal epithelium, the contribution of this practice to the transmission and acquisition of STIs including HIV may be important.

The objective of this study was to examine specific content and types of products used for rectal douching among men and women (including both commercial and non-commercial, “homemade” products) and to evaluate rectal douching practices and factors associated with douching. We hypothesized that there would be variation in both commercial and “homemade” products used for rectal douching and that factors associated with rectal douching would vary by age, gender, and sexual behaviors. We further hypothesized that rectal douching would be associated with self-reported STI status including HIV.

## Methods

### Study population and design

We conducted a cross-sectional study, using an internet-based survey (see Additional file 1) to collect information on rectal douching practices including information on products and substances used for douching. Women and men who were at least 18 years of age and reported receptive AI in the past 3 months were eligible to complete the survey. The study was approved by the Human Subjects Committee at the University of California Los Angeles.

Recruitment was conducted by the International Rectal Microbicide Advocates (IRMA; http://www.rectalmicrobicides.org), a network of over 1,200 advocates, policy makers and scientists from over 60 countries working to advance rectal microbicide research. Participants were recruited through brief email messages sent by IRMA through various topical, regional, and community listservs (i.e. electronic mailing lists). The listservs primarily included those focused on HIV, microbicides, gay men’s health, women’s health, and sexual and reproductive health. In addition, several websites posted information and links to the survey, including sites targeted to gay men and rectal microbicides.

### Data collection

Those interested in participating were directed to the IRMA website, which contained a link to the study survey. All participants provided electronic informed consent before starting the study questionnaire, which took approximately 15 minutes to complete. No remuneration was given for participation. The self-administered web-based survey was offered in multiple languages including English, French, German, Mandarin, Portuguese, Russian, Spanish, and Thai. Translated questionnaires were pilot tested with native speakers in order to ensure comprehension of the translated materials.

Participants were recruited over a 10-month period from August 2011 – May 2012. In addition to basic demographic information, the survey included questions on sexual behaviors, history of STIs, and practices surrounding AI including douching. Respondents were asked about frequency, reasons, timing (i.e., before and/or after anal intercourse), and the type(s) of rectal douches/enemas used. Specifically, those who reported rectal douching in the past 3 months used a 5-point Likert scale to respond to the question “How often did you use an enema or douche rectally before having receptive AI (*you had a penis in your butt/bum*)?” Likewise, a similar question assessed rectal douching after receptive AI. The survey also included an image-based list of douches available commercially. While efforts were made to include images of douches available globally, the majority of images were based on products available in the United States and Canada. Respondents were asked to select products from the image list or specify commercial products (if not listed on image list) used in the past 3 months. Questions regarding the use of non-commercial or “homemade” douches had the following answer choices: water, water with salt, water with soap, alcohol, or the option to specify a product not listed.

### Statistical analysis

Descriptive statistics were calculated for the total sample and by rectal douching status, comparing those who reported rectal douching to those who did not. Differences between groups were evaluated using chi-square methods for categorical variables and t-tests (or Kruskal-Wallis test where appropriate) for continuous variables. Associations between reported douching practices and other factors including age and reported STI history were evaluated using logistic regression analysis. All analyses were conducted using SAS version 9.2 (SAS Institute Inc., Cary, NC, USA).

## Results

### Sample characteristics

Among the 2,436 respondents who attempted the survey, 70.8% (n = 1,725) were eligible and included in the study. Respondents represented 112 countries with half from North America (55%), nearly a quarter from Europe (22%), as well as Latin America (14%), Asia (5%), and Africa (3%) (Table 1). Furthermore, the majority of respondents were male (88%) and less than 40 years of age (mean age: 36.5 years; range: 18–87 years).

**Table 1:** Characteristics of respondents in the international

	**n**	**%**
***Demographic characteristics***		
Age, years^		36.5 (11.6)
Male	1,514	87.7
Region		
African	45	2.6
Asia	90	5.2
Europe	370	21.5
Latin America/Caribbean	237	13.7
North America	944	54.7
Other	39	2.3
***Sexual behaviors***		
Gender of sex partners, past 3 months		
MSM	1,422	82.3
MSM/W	92	5.3
WSM	162	9.4
WSM/W	49	2.8
Types of sex partners, past 3 months		
Main or regular partner	1,125	65.2
Casual partner	770	44.6
Anonymous partner	324	18.8
Trade/transactional partner	88	5.1
Number of partners, past 3 months^^		3(1–250)
Always use condom for RAI, past 3 months	589	34.9

Abbreviations. *MSW* Men who have sex with women, *MSM/W* Men who have sex with men and women, *WSM* Women who have sex with men, *WSM/W* Women who have sex with men and women, *RAI* Receptive anal intercourse.

^Data represents mean and standard deviation.

^^Data represent median and range.

Rectal douching survey, Aug 2011-May 2012 (n = 1,725).

### Frequency and reasons for rectal douching/enema use

Among the 1,725 respondents, 62% (n = 1,070) reported rectal douching/enema use before or after AI, with the majority reporting douching before AI (83% douching always or most of the time) and fewer reporting douching after AI (16% douching always or most of the time) (Table 2). In exploring the prevalence of rectal douching before and after AI, we found that among respondents who reported douching before AI ‘always’ or ‘most of the time’, 19% (176/885) also reported doing so after AI. Almost all those who reported rectal douching before AI reported cleanliness as the reason for douching with others reporting AI as more pleasurable (62%) or sex partner’s preference (18%) as the main reason for douching. Among those who did not report any rectal douching/enema use (n = 655), the most common reason noted was that it was unnecessary (38%), they didn’t know about rectal douches (27%), or they did not have access to douches/enemas (27%).

**Table 2:** Rectal douching behaviors among respondents in the international

	**n**	**%**
Rectal douching/enema use, past 3 months	1,070	62.3
Frequency of rectal douching/enema use before anal intercourse*		
Always	526	49.3
Most of the time	360	33.7
Some of the time	151	14.2
Never	30	2.8
Frequency of rectal douching/enema use after anal intercourse*		
Always	89	8.4
Most of the time	87	8.2
Some of the time	205	19.3
Never	685	64.3
Reasons for rectal douching/enema use*		
Cleanliness/hygiene	983	94.9
Anal intercourse more pleasurable	655	63.2
Partner’s preference/request	198	19.1
Reasons for no rectal douching/enema use**		
Unnecessary	246	37.6
Didn’t know about rectal douches/enemas	175	26.7
Didn’t have access	177	27.0
Dislike	103	15.7
No time	79	12.1
***Rectal douche type/content***		
Commercial products	332/1070	31.0
Laxative-based	62/332	18.7
Mineral oil/glycerin-based	73/332	22.0
Saline-based	187/332	56.3
Sodium-phosphate	107/332	32.2
Other product	49/332	14.8
Non-commercial, ‘homemade’ products	990/1070	92.5
Water	809/990	81.6
Water + salt	47/990	4.8
Water + soap	113/990	11.4
Alcohol	12/990	1.2
Other	35/990	3.5

Rectal douching survey, Aug 2011-May 2012 (n = 1,725).

*Among those who reported rectal douching/enema use (n = 1,070).

**Among those who reported no rectal douching/enema use (n = 655).

### Types of rectal douches/enemas used

Commercial products were used by 31% of respondents. The most common products used were saline-based products (56%), with a smaller minority reporting the use of laxative-based and mineral oil rectal douches/enemas (Table 2). However, the majority of rectal douchers reported using non-commercial/homemade products (93%), with 75% using homemade products exclusively (i.e., no commercial products). Water was the most common product reported (82%), while other less prevalent non-commercial products included water and soap (11%), water and salt (5%), and alcohol such as wine (1%). A small minority of respondents reported on other products such as lemon juice, urine, vinegar, and coffee (<1% for each). Shower head hose and nozzle or a “sinker” (a portable rubber or vinyl hose that attaches to a sink) were the most common type of douching equipment used with non-commercial products, with 50% of those who reported douching with non-commercial products reporting its use. In contrast, plastic bottles such as water bottles or other containers not made for rectal douching were less common (12.4%), though use among those who reported this type of equipment was non-trivial with the average use being 6 times in the past 3 months.

### Factors associated with rectal douching/enema use

The prevalence of rectal douching varied by demographic characteristics and sexual risk behaviors (Table 3). Those who reported rectal douching/enema use were slightly older (mean age 38.1 years vs. 34.0 years; p value < .01) and more likely to be male (70% vs. 32%; p value < .01). The prevalence of rectal douching also varied by region, with the highest prevalence in Europe and North America (72% and 71% respectively) and the lowest prevalence in Latin America/Caribbean (40%; p value < .01). Rectal douching was also higher among those who reported having receptive AI more frequently, lubricant use for receptive AI, substance use with sexual activity, being HIV-positive, and those reporting a history of sexually transmitted infections (STIs) in the past year including rectal chlamydia, gonorrhea, and syphilis. In multivariable analyses, after adjusting for age, gender, region, and condom use, factors independently associated with rectal douching included lubricant use (adjusted odds ratio [AOR] = 1.77, 95% confidence interval [CI] = 1.10, 2.85), substance use with sexual activity (AOR = 1.93, 95% CI = 1.50, 2.49), and self-reported history of an STI in the past 12 months (AOR = 1.74, 95% CI = 1.01, 3.00).

**Table 3:** Prevalence and factors associated with rectal douching/enema use by demographic characteristics and sexual behaviors among respondents in the international rectal douching survey, May 2011-August 2012 (n = 1,725)

		**Rectal Douche/enema use**		**Adjusted OR**	**95% CI**
	**n**	**%**	**p value**		
Age, years*			<.01	1.03	(1.02–1.04)
Douchers		38.1 (11.7)			
Non-Douchers		34.0 (10.8)			
Gender			<.01		
Male	1,258	70.4		1.00	Reference
Female	81	32.4		0.22	(0.15–0.32)
Region			<.01		
Africa	37	61.8		0.41	(0.18–0.84)
Asia	58	52.7		0.39	(0.18–0.84)
Europe	328	71.9		0.92	(0.67–1.26)
Latin America/Caribbean	107	39.9		0.21	(0.14–0.31)
North America	773	70.7		1.00	Reference
Other	36	72.0		1.57	(0.56–4.40)
No. of times, receptive anal intercourse, past 3 months**			<.01	1.01	(1.00–1.02)
Douchers		6 (3–15)			
Non-Douchers		4 (2–10)			
Always use condoms for RAI, past 3 months			0.08		
Yes	414	63.8		0.88	(0.68–1.15)
No	817	67.9		1.00	Reference
Lubricant use for RAI, past 3 months			<.01		
Yes	1,159	67.8		1.77	(1.10–2.85)
No	71	50.0		1.00	Reference
Substance use, with sexual activity			<.01		
Yes	499	73.6		1.93	(1.50–2.49)
No	365	46.1		1.00	Reference
STI, past 12 months			<.01		
Yes	170	69.4		1.74	(1.01–3.00)
No	688	57.1		1.00	Reference
HIV-positive			<.01		
Yes	352	72.4		1.68	(1.26–2.24)
No	499	52.5		1.00	Reference

Abbreviations. *OR* odds ratio, *RAI* receptive anal intercourse, *STI* Sexually transmitted infection.

*Data represent mean and standard deviation.

**Data represent median and interquartile range.

**Figure 1:**
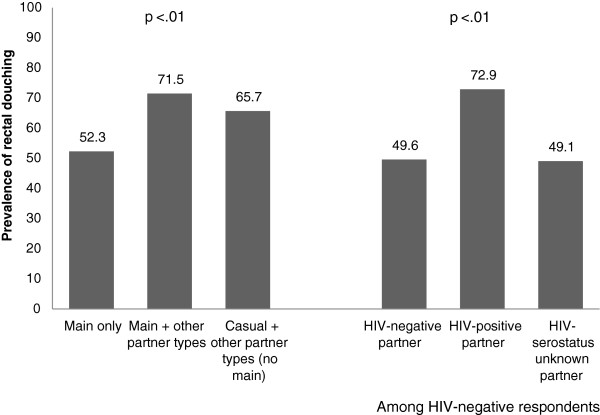
Prevalence of rectal douching, by partnership type and partner HIV-status among respondents in the international rectal douching survey, May 2011-August 2012 (n = 1,725).

Exploring partnership specific factors revealed that the prevalence of rectal douching was higher in the context of non-main partnerships (Figure 1). Specifically, those who reported having only a main/regular sexual partner in the previous three months had a 52% prevalence of rectal douching as compared to 66% among those who reported having casual partnerships including one-time and anonymous partners (but no main partnership; p < .01). Not surprisingly, consistent condom use for receptive AI in the context of main partnerships was low (29%), though remained relatively low even in the context of casual partnerships (44%) (data not shown). Furthermore, in limiting our analyses to those who were HIV-negative (i.e., at risk for HIV-acquisition) we found that the prevalence of rectal douching was highest among those who reported having an HIV-seropositive partner (Figure 1).

## Discussion

Based on this internet survey, we found that a substantial proportion of respondents reported rectal douching before receptive AI with a non-trivial proportion reporting rectal douching after receptive AI. These findings are consistent with the small number of studies conducted on this topic and suggest that rectal douching with AI is a relatively common practice [18,22]. However, our study is one of the first to report on the content and type of rectal douches used for AI. While water enemas – the most commonly reported product in this study – are hypotonic and have fewer reported complications when compared to hyperosmolar enemas, they have been associated with rectal epithelium loss and damage when compared to isotonic solutions such as polyethylene glycol [24-27]. Likewise, colonic irritation, colitis, and rectal epithelium damage has been noted with some of the other products used for rectal douching including water and soap, sodium phosphate enemas, and laxative-based enemas (e.g., bisacodyl) [24,28,29]. Given that the most commonly used products may cause damage to the rectal epithelium, this practice may increase the risk of transmission and acquisition of STIs/HIV. Furthermore, these findings suggest that harm reduction strategies recommending products that minimize rectal epithelial damage may be warranted.

Our finding that douching varied by region is supported by the small number of rectal douching prevalence studies [18,22,23] as well as other studies on rectal practices surrounding AI such as lubricant use [30] and may reflect variations in sexual practices by region. Indeed, in exploring reasons for lack of rectal douching by region we found that in regions where the prevalence of rectal douching was low such as Asia, reporting that ‘Didn’t know people used an enema or douche for anal intercourse’ was far more common than regions where rectal douching was high (53% in Asia vs. 17% in North America, p value < .01; data not shown). Consequently, the impact of any harm reduction strategies to reduce the use of potentially harmful products may be more relevant in regions where this practice is more pervasive.

We also found that the prevalence of rectal douching varied by a number of sexual risk behaviors including substance use. Specifically, more substance users reported rectal douching as compared to non-users. This may be partly explained by evidence which suggests that substance use, in particular methamphetamine use is associated with prolonged sexual encounters, including an increase in number of events with casual or anonymous partners [31]. Moreover, certain substances including opioids increase the likelihood of bowel dysfunction and constipation, potentially increasing the need for rectal douching [32-34]. Beyond sexual risk behaviors, rectal douching was also associated with sexual health outcomes including HIV. Our finding that rectal douching was more prevalent among those who are HIV-positive is supported by a number of studies that have shown that HIV-status is associated with rectal douching [16-19]. Furthermore, our results indicate that a history of STIs in the past year, including rectal chlamydia, gonorrhea, and syphilis was also associated with rectal douching even after adjusting for potential confounders such as condom use, substance use with sex, and HIV-status. This not only adds to the small number of studies which have noted an association with rectal douching and non-HIV STIs including LGV and HBV, but also lends epidemiologic support to the hypothesis that rectal products used for anal intercourse may facilitate transmission of STIs.

Rectal douches/enemas may serve as a possible delivery mechanism for rectal microbicides, which are currently under development [35]. Findings from this study add support to the promise of the acceptability of this delivery method, given that the behavior is already commonly practiced before receptive AI [36,37]. Of note is our finding of the association between rectal douching and other behaviors associated with risk of acquisition of STIs/HIV, such as substance use that suggest use of HIV prevention via rectal douches may fit into the repertoire of those most at risk and when engaging in their riskiest behaviors. Furthermore, our findings that the prevalence of douching is higher in the context of casual partnership including one-time and anonymous partnerships, as well as serodiscordant partnerships, suggest that those most at risk or during periods of greatest risk are also most likely to practice douching. These factors along with the potential for the improved safety profile of a rectal microbicide over existing commonly used products, suggests that douches could hold great potential as delivery mechanisms for event-based methods of prevention. Given past challenges with adherence to topical microbicides [38,39], another delivery method that is part of the behavioral repertoire of many people who engage in AI may enhance acceptability and therefore, adherence.

A number of limitations related to this study should be noted. The survey respondents represent a convenience sample drawn from a larger population of users of the targeted email lists, chat rooms, and websites and it is unknown what proportion of subscribers completed the survey. This limitation of online sampling has been previously noted; however, the strength of this method is the ability to access hard-to-reach groups and eliminate some of the validity issues associated with interview-based data on sensitive sexual behaviors [40,41]. Furthermore, interpretation of the association between STIs and rectal douching is limited by the fact that STI status is based on self-report and includes events occurring in the past year, while rectal douching practices relate to those reported for the past 3 months. However, evidence that rectal douching behaviors may be pervasive and start at a young age increases the likelihood that practices in the past 3 months may be indicative of rectal douching practices overall [18].

## Conclusion

In summary, rectal douching for receptive AI is common and because rectal douching was associated with other sexual risk behaviors, the contribution of this practice to the transmission and acquisition of STIs including HIV may be important. While further longitudinal studies may help to further delineate associations between STIs/HIV and the different rectal douching products used, this study provides important information for the promotion of better rectal safety and rectal health.

## Competing interests

The authors declare that we have no competing interests.

## Authors’ contributions

MJ: study conception and design, data collection, supervision of data analysis, drafting of manuscript; SS: data collection, data analysis, and drafting of manuscript; MA and JP: study conception, data collection, and interpretation of results. PM: study conception and design, data interpretation, and manuscript preparation. All authors read and approved the final manuscript.

## Pre-publication history

The pre-publication history for this paper can be accessed here:

http://www.biomedcentral.com/1471-2334/14/95/prepub

## Supplementary Material

Additional file 1 Rectal Douching and Enema Survey. Click here for file
