# Supplementary material for: Prevalence and types of rectal douches used for anal intercourse: results from an international survey
Source: BMC Infect Dis. 2014 Feb 21;14:95. doi: 10.1186/1471-2334-14-95 (PMC4015843; doi:10.1186/1471-2334-14-95)
Supplement: Additional file 1 — Rectal Douching and Enema Survey. [file 1471-2334-14-95-S1.pdf]

Rectal Douching and Enema Survey

The International Rectal Microbicide Advocates (IRMA) and researchers at the University of California, Los Angeles (UCLA) School of Public Health are conducting a brief survey to help us better understand the types of products people use rectally for anal sex including lubricants and enemas or douches. We are trying to gain a better understanding of rectal practices and behaviors that may affect the risk for sexually transmitted infections among people who practice anal intercourse. We hope you will fill out this brief anonymous survey (estimated time to complete: less than 15 minutes).

In order to decide whether or not you would like to be part of this study, you should understand enough about the study to make an informed judgment. Detailed information about this study is located here. Once you have reviewed this information, if you still wish to participate, please proceed to the survey. This is an anonymous survey and all responses will be held in the strictest confidence.

1. Do you identify as:

   Male
   Female
   Transgender, Male to Female
   Transgender, Female to Male

2. How old are you? [please enter your age in years]

3. Country where you live? [please select one…]

4. What race/ethnicity do you identify as?

   Asian
   Black
   Hispanic
   White
   Other

5. We would like to ask you some general questions about your sexual behaviours in the past 3 months

6. In the past 3 months, have you had anal intercourse (in other words did you put your penis into someone's butt/bum or did they put their penis inside your butt/bum)?

   No, I did not have anal intercourse in the past 3 months
   Yes, I only had receptive anal intercourse in the past 3 months (meaning someone's penis inside your butt/bum)
   Yes, I only had insertive anal intercourse in the past 3 months (meaning you put your penis inside someone's butt/bum)
   Yes, I had both receptive and insertive anal intercourse in the past 3 months

7. In the past 3 months, did you use an enema or douche rectally? An enema or douche is a liquid, such as water, that you put inside your rectum and then expel.

Yes
No

8. We are interested in reasons why people may or may not use enemas or douches rectally. You responded that in the past 3 months you did not use an enema or douche rectally. Could you please tell us why? [please select all that apply]

   Didn't know people used an enema or douche for anal intercourse
   Unnecessary
   Not enough time
   Dislike
   Didn't have access to enema or douche
   Only had insertive anal intercourse (was the top partner) during the past 3 months
   Other

9. In the past 3 months, have you had sex with men, women, or both?

   Men only
   Women only
   Both Men and Women

10. In the past 3 months, what type of sexual partners have you had? Please select all that apply.

    Main or regular partner
    Casual partner
    One time partner
    Anonymous partner
    Trade partner (Someone who you had sex with for money, drugs or other goods or someone who had sex with you for money, drugs or other goods.)

11. Please enter your best estimate of the number of times in the past 3 months you had receptive anal intercourse (you had a penis in your butt/bum)? If you did not have receptive anal intercourse in the past 3 months please enter 0.

    Number of times

12. Now we'd like to ask you about commercial lubricants as well as enemas or douches that you may have used in your rectum (butt/bum).

By commercial lubricant we mean a lubricant designed specifically for sexual intercourse that can be purchased in a store or on-line. Do NOT count products such as hand lotion, Crisco, saliva, or the lubricant on pre-lubricated condoms when answering this question.

An enema or douche is a liquid, such as water, that you put inside your rectum and then expel. Some people use an enema or douche before or after they have anal intercourse.

Please tell us if you experienced any of the following in the past 3 months and the past month.

13. When you had receptive anal intercourse (had a penis in your butt/bum), how often did your partner use a condom?

    Past 3 months

    Always (100% of the time)
    Most of the time (75-99% of the time)
    Often (50-74% of the time)
    Sometimes (25-49% of the time)
    Rarely (1-24% of the time)
    Never

    Past month

    Always (100% of the time)
    Most of the time (75-99% of the time)
    Often (50-74% of the time)
    Sometimes (25-49% of the time)
    Rarely (1-24% of the time)
    Never
    I did not have receptive anal intercourse (was not a BOTTOM) in the last month

14. When you had receptive anal intercourse (had a penis in your butt/bum), how often did you use a lubricant (lube) designed specifically for sex that can be purchased in a store or on-line?

    Past 3 months

    Always (100% of the time)
    Most of the time (75-99% of the time)
    Often (50-74% of the time)
    Sometimes (25-49% of the time)
    Rarely (1-24% of the time)
    Never

    Past month

    Always (100% of the time)
    Most of the time (75-99% of the time)

Often (50-74% of the time)
Sometimes (25-49% of the time)
Rarely (1-24% of the time)
Never
I did not have receptive anal intercourse (was not a BOTTOM) in the last month

15. How often did you use an enema or douche rectally ***before*** having receptive anal intercourse (you had a penis in your butt/bum)?

    Past 3 months

    Always (100% of the time)
    Most of the time (75-99% of the time)
    Often (50-74% of the time)
    Sometimes (25-49% of the time)
    Rarely (1-24% of the time)
    Never

    Past month

    Always (100% of the time)
    Most of the time (75-99% of the time)
    Often (50-74% of the time)
    Sometimes (25-49% of the time)
    Rarely (1-24% of the time)
    Never
    I did not have receptive anal intercourse (was not a BOTTOM) in the last month

16. How often did you use an enema or douche rectally ***after*** having receptive anal intercourse (you had a penis in your butt/bum)?

    Past 3 months

    Always (100% of the time)
    Most of the time (75-99% of the time)
    Often (50-74% of the time)
    Sometimes (25-49% of the time)
    Rarely (1-24% of the time)
    Never

    Past month

    Always (100% of the time)
    Most of the time (75-99% of the time)
    Often (50-74% of the time)
    Sometimes (25-49% of the time)
    Rarely (1-24% of the time)
    Never
    I did not have receptive anal intercourse (was not a BOTTOM) in the last month

17. We are interested in reasons why people may or may not use enemas or douche rectally. You responded that you have used an enema or douche rectally ***before*** having receptive anal intercourse (you had a penis in your butt/bum). Could you please tell us why? [please select all that apply]

    Constipation
    Cleanliness/Hygiene
    Make anal intercourse more pleasurable
    Partner prefers
    Other

18. We are interested in reasons why people may or may not use enemas or douche rectally. You responded that at least some of the time you did not use an enema or douche rectally ***before*** having receptive anal intercourse (you had a penis in your butt/bum). Could you please tell us why? [please select all that apply]

    Unnecessary
    Not enough time
    Dislike
    Didn't have access to enema or douche
    Other

19. We are interested in reasons why people may or may not use enemas or douche rectally. You responded that you have used an enema or douche rectally ***after*** having receptive anal intercourse (you had a penis in your butt/bum). Could you please tell us why? [please select all that apply]

    Constipation
    Cleanliness/Hygiene
    Make anal intercourse more pleasurable
    Partner prefers
    Other

20. We are interested in reasons why people may or may not use enemas or douche rectally. You responded that at least some of the time you did not use an enema or douche rectally ***after*** having receptive anal intercourse (you had a penis in your butt/bum). Could you please tell us why? [please select all that apply]

    Unnecessary
    Not enough time
    Dislike
    Didn't have access to enema or douche
    Other

21. *[Hidden Question]*

22. Please look at the following commercial products used rectally as enemas or douches and tell us about which products you used in the **past 3 months** and then scroll down to answer some questions regarding these products.

23. Which one of these products did you use most often?

Past 3 months

I did not use any commercial products
Commercial product other than the ones above
American Fare Enema – Sodium Phosphate
Chester Gent-L Tip Enema – Mineral Oil
Chester Gent-L Tip Enema – Sodium Phosphate
CVS Enema – Mineral Oil
CVS Enema – Sodium Phosphate Latex Free
Docusol Mini Enema – Docusate
Enemeez Mini Enema – Docusate
Equate Enema – Sodium Phosphate
Fleet Enema – Bisacodyl
Fleet Enema – Glycerin
Fleet Enema – Mineral Oil
Fleet Enema – Naturals with Aloe
Fleet Enema – Saline Extra
Fleet Enema – Saline
Medline Enema – Mineral Oil
Medline Enema – Sodium Phosphate
Quality Choice Enema – Saline
Ready to Use Enema – Saline
Rugby Laboratories Enema – Saline
Sata Minica-s Enema – Laxative
Walgreens Enema – Saline Laxative

If you used a commercial product other than the ones pictured above in the past 3 months, please specify:

24. How many times did you use this enema or douche? If you don't remember please enter 999.

    Past 3 months

25. Which one of these products did you use the second most often?

    Past 3 months

I did not use any commercial products
Commercial product other than the ones above
American Fare Enema – Sodium Phosphate
Chester Gent-L Tip Enema – Mineral Oil
Chester Gent-L Tip Enema – Sodium Phosphate
CVS Enema – Mineral Oil
CVS Enema – Sodium Phosphate Latex Free
Docusol Mini Enema – Docusate
Enemeez Mini Enema – Docusate
Equate Enema – Sodium Phosphate
Fleet Enema – Bisacodyl
Fleet Enema – Glycerin
Fleet Enema – Mineral Oil

Fleet Enema – Naturals with Aloe
Fleet Enema – Saline Extra
Fleet Enema – Saline
Medline Enema – Mineral Oil
Medline Enema – Sodium Phosphate
Quality Choice Enema – Saline
Ready to Use Enema – Saline
Rugby Laboratories Enema – Saline
Sata Minica-s Enema – Laxative
Walgreens Enema – Saline Laxative

If you used a commercial product other than the ones pictured above in the past 3 months, please specify:

26. How many times did you use this enema or douche? If you don't remember please enter 999.

    Past 3 months

27. Please choose any other types of products that you used in the past 3 months as enemas or for douching your butt/bum. Again, please refer to the list of products provided to you and choose up to 10 products.

    Product 1
    Product 2
    Product 3
    Product 4
    Product 5
    Product 6
    Product 7
    Product 8
    Product 9
    Product 10

    American Fare Enema – Sodium Phosphate
    Chester Gent-L Tip Enema – Mineral Oil
    Chester Gent-L Tip Enema – Sodium Phosphate
    CVS Enema – Mineral Oil
    CVS Enema – Sodium Phosphate Latex Free
    Docusol Mini Enema – Docusate
    Enemeez Mini Enema – Docusate
    Equate Enema – Sodium Phosphate
    Fleet Enema – Bisacodyl
    Fleet Enema – Glycerin
    Fleet Enema – Mineral Oil
    Fleet Enema – Naturals with Aloe
    Fleet Enema – Saline Extra
    Fleet Enema – Saline
    Medline Enema – Mineral Oil
    Medline Enema – Sodium Phosphate

Quality Choice Enema – Saline
Ready to Use Enema – Saline
Rugby Laboratories Enema – Saline
Sata Minica-s Enema – Laxative
Walgreens Enema – Saline Laxative

28. Please look at the following commercial products used rectally as enemas or douches and tell us about which products you used in the past month and then scroll down to answer some questions regarding these products.

29. Which <u>one</u> of these products did you use <u>most often</u>?

    Past month

    I did not use any commercial products
    Commercial product other than the ones above
    American Fare Enema – Sodium Phosphate
    Chester Gent-L Tip Enema – Mineral Oil
    Chester Gent-L Tip Enema – Sodium Phosphate
    CVS Enema – Mineral Oil
    CVS Enema – Sodium Phosphate Latex Free
    Docusol Mini Enema – Docusate
    Enemeez Mini Enema – Docusate
    Equate Enema – Sodium Phosphate
    Fleet Enema – Bisacodyl
    Fleet Enema – Glycerin
    Fleet Enema – Mineral Oil
    Fleet Enema – Naturals with Aloe
    Fleet Enema – Saline Extra
    Fleet Enema – Saline
    Medline Enema – Mineral Oil
    Medline Enema – Sodium Phosphate
    Quality Choice Enema – Saline
    Ready to Use Enema – Saline
    Rugby Laboratories Enema – Saline
    Sata Minica-s Enema – Laxative
    Walgreens Enema – Saline Laxative

If you used a commercial product other than the ones pictured above in the <u>past month</u>, please specify:

30. How many times did you use this enema or douche? If you don't remember please enter 999.

    Past month

31. Which <u>one</u> of these products did you use the <u>second most often</u>?

    Past month

    I did not use any commercial products
    Commercial product other than the ones above

American Fare Enema – Sodium Phosphate
Chester Gent-L Tip Enema – Mineral Oil
Chester Gent-L Tip Enema – Sodium Phosphate
CVS Enema – Mineral Oil
CVS Enema – Sodium Phosphate Latex Free
Docusol Mini Enema – Docusate
Enemeez Mini Enema – Docusate
Equate Enema – Sodium Phosphate
Fleet Enema – Bisacodyl
Fleet Enema – Glycerin
Fleet Enema – Mineral Oil
Fleet Enema – Naturals with Aloe
Fleet Enema – Saline Extra
Fleet Enema – Saline
Medline Enema – Mineral Oil
Medline Enema – Sodium Phosphate
Quality Choice Enema – Saline
Ready to Use Enema – Saline
Rugby Laboratories Enema – Saline
Sata Minica-s Enema – Laxative
Walgreens Enema – Saline Laxative

If you used a commercial product other than the ones pictured above in the past month, please specify:

32. How many times did you use this enema or douche? If you don't remember please enter 999.

    Past month

33. In the *past 3 months* did you use anything else in your butt/bum as an enema or douche that you did not purchase in a store but made at home? If so, please check all that apply:

    No I did not use anything else
    Water only
    Water with salt
    Water with soap
    Alcohol such as wine
    Lemon juice
    Other

34. Which one of these products did you use most often in the past 3 months? (Choose one from the list of products)

    Water only
    Water with salt
    Water with soap
    Alcohol such as wine
    Lemon juice
    Other

35. How many times did you use this product in the past 3 months? *enter 999 if you can't remember or prefer not to answer*

times

36. Which one of these products did you use second most often in the past 3 months? (Choose one from the list of products)

Water only
Water with salt
Water with soap
Alcohol such as wine
Lemon juice
Other

37. How many times did you use this product in the past 3 months? *enter 999 if you can't remember or prefer not to answer*

times

38. Which one of these products did you use most often in the past month? (Choose one from the list of products)

Water only
Water with salt
Water with soap
Alcohol such as wine
Lemon juice
Other

39. How many times did you use this product in the past month? *enter 999 if you can't remember or prefer not to answer*

times

40. Which one of these products did you use second most often in the past month? (Choose one from the list of products)

Water only
Water with salt
Water with soap
Alcohol such as wine
Lemon juice
Other

41. How many times did you use this product in the past month? *enter 999 if you can't remember or prefer not to answer*

times

42. There are different ways to use an enema or douche rectally including sink and shower-head hose attachments and over-the-counter kits. In the following questions, please tell us about the type of enema or douche kits you have used.

    please enter 0 if you did not use this type of enema or douche
    please enter 999 if you can't remember or prefer not to answer

43. When you used an enema or douche in your butt/bum, how many times did you use a non-disposable douche or enema bag system (neoprene or rubber bag, rubber hose, plastic clamp, and plastic or rubber nozzle)?

    Past 3 months
    Past month

44. When you used an enema or douche in your butt/bum, how many times did you use a shower head hose and nozzle or a "sinker" (a portable rubber or vinyl hose that attaches to a sink)?

    Past 3 months
    Past month

45. When you used an enema or douche in your butt/bum, how many times did you use an over-the-counter disposable enema product (e.g., Fleet®)?

    Past 3 months
    Past month

46. When you used an enema or douche in your butt/bum, how many times did you use a re-useable bulb enema?

    Past 3 months
    Past month

47. When you used an enema or douche in your butt/bum, how many times did you use a plastic bottle or other container not made for douching/enemas to do this (e.g., water bottle)?

    Past 3 months
    Past month

48. The *last time* you used an enema or douche rectally, on average how long did you keep the solution in your butt/bum before expelling it?

    # minutes

49. We have a few last questions to ask you.

50. In the past 12 months, how many people have you had

vaginal intercourse with?
Please enter number of people
anal intercourse with?
Please enter number of people

51. In the past 12 months, what types of drug(s), if any, have you used when you had sexual activity? Please select all that apply.

No drugs used
Crystal/Methamphetamine
Ecstasy
Poppers
Ketamine
GHB
Cocaine
Heroin
Marijuana
Viagra, Cialis or Levitra
Other

52. In the past 12 months, has anyone paid you or have you paid for sex with money, drugs, or anything else?

Yes
No

53. In the past 12 months have you had sex with an HIV-positive partner

Yes
No
Don't know

54. Have you *ever* tested positive for HIV?

Yes
No
Don't know

55. In the past 12 months, has a doctor, clinician, or health care provider told you that you have a sexually transmitted infection?

Yes
No
Don't know

56. Which sexually transmitted infection did you have? Please select all that apply

Chlamydia
Rectal Chlamydia
Genital Warts (HPV, Human Papillomavirus)
Rectal or anal warts (HPV, Human Papillomavirus)
Gonorrhea
Rectal Gonorrhea
Genital Herpes
Syphilis
Other (please specify below)

57. Which "other" sexually transmitted infection did you have?

Based on your response you may not be eligible to complete this survey. We appreciate your interest and thank you for your time.

Thank you for taking this survey. Your input is very much appreciated.
